# Supplementary material for: Parallel sequencing lives, or what makes large sequencing projects successful
Source: Gigascience. 2017 Oct 18;6(11):1–6. doi: 10.1093/gigascience/gix100 (PMC5714127; doi:10.1093/gigascience/gix100)
Supplement: Additional Files [file gix100_supp.zip › additional_file03.pdf]

Why T47D_rep2 and b1913e6c1_51720e9cf are not singletons

The stories of T47D_rep2 and b1913e6c1_51720e9cf reflect challenges associated to managing and analyzing the growing amount of sequencing data (**Table 1**). Some of these challenges are not new or exclusive of high throughput sequencing data and partly reflect suboptimal habits (e.g. poor description of samples, unsystematic sample naming, untidy data organisation and undocumented procedures) that have been just aggravated by the rapid spread of high throughput sequencing. For instance, in cross-sectional population studies, samples are normally collected at the same time and analyzed jointly, which may make more obvious the need to define sample naming schemes and to systematically collect the metadata required for the analysis. Conversely, in most research groups, sequencing experiments are performed independently by several people, accumulate over longer periods of time and are not initially meant to be analyzed together.

In addition, the arrival of a technology that requires informatics skills into a historically wet lab-based field often generates situations in which those who perform the experiments are not aware of the computational challenges of the analysis. In this sense, working groups that are relatively small and/or have limited computational infrastructures are more prone to suffer from them. In contrast to large-scale data-intensive projects, which are more likely to allocate resources to anticipate, avoid and fix such issues; for instance, the 4DNucleome Project has established formal working groups employing tens of scientists responsible for the data standards and analysis protocols [1].

Nevertheless, the problems we list are present to some extent in larger scale initiatives too. For instance, in the SRA [2] repository there are ~30,000 experiments (32 Terabases) with an ‘unspecified’ instrument (**Additional file 4**). Also in the SRA repository [2], only for the top 25 submitter institutions there are several Petabases of data assigned to multiple entries probably referring to the same submitter (**Additional file 5**). Altogether, this represents a large amount of data that will be overlooked in many searches, which could have been avoided by enabling mandatory fields with predefined vocabulary. For another example, the ENCODE consortium published mislabelled or failed experiments [3], and approximately 20% of the uploaded ChIP-seq profiles correlate more with a negative control than with their replicate (unpublished observation).

References

1. Dekker J, Belmont AS, Guttman M, Leshyk VO, Lis JT, Lomvardas S, et al. The 4D Nucleome Project. bioRxiv. 2017.

2. Leinonen R, Sugawara H, Shumway M, International Nucleotide Sequence Database Collaboration. The sequence read archive. Nucleic Acids Res. 2011;39 Database issue:D19-21. doi:10.1093/nar/gkq1019.

3. Cuscó P, Filion GJ. Zerone: a ChIP-seq discretizer for multiple replicates with built-in quality control. Bioinformatics. 2016;32:2896–902. doi:10.1093/bioinformatics/btw336.
