# Supplementary material for: Parallel sequencing lives, or what makes large sequencing projects successful
Source: Gigascience. 2017 Oct 18;6(11):1–6. doi: 10.1093/gigascience/gix100 (PMC5714127; doi:10.1093/gigascience/gix100)
Supplement: Additional Files [file gix100_supp.zip › additional_file06.pdf]

Considerations and costs of implementing and maintaining a DATA lab culture

In the manuscript we propose actions to achieve a lab culture of Documentation, Automation, Traceability and Autonomy (DATA) and compliance with the FAIR Principles [1]. Here we comment on the considerations and costs of implementing and maintaining these actions based on our own experience. For simplicity, we will consider an scenario in which there are experimentalists and analysts, where one analyst acts as the data manager.

Metadata collection

Implementing a metadata collection system requires several considerations.

1. What metadata are collected? All experimentalists, analysts and the data manager need to be involved at this stage and anticipate what information is required to both describe a sequencing experiment and analyze the derived data. Consequently, this action is costly in terms of time for all members of the lab. In addition, it requires meeting a compromise between the brevity of the metadata to achieve user acceptance and the depth of metadata to achieve the described positive impact.
2. How are metadata collected? In our projects we use Google Forms and Sheets because they are free as well as easy to use and implement. Nonetheless, we are aware that some groups working with sensitive human data may be reluctant to use third-party systems and may need to opt for systems that are not free, less intuitive and that have a heavier implementation load; in addition, we see an inherent confrontation between the compliance of the FAIR Principles [1] and the availability of protected human data. Whatever the software used to collect the metadata is, an important consideration is the choice of drop-down lists or free-text fields, and how this contributes to the completeness and parsability of the metadata. We recommend using drop-down lists as much as possible to minimize the presence of typos and variability in the metadata (e.g. the species written

as a mix of “human”, “Human”, “Homo sapiens” is undesired). Even for fields in which free text would seem the best option a priori, we recommend specifying predefined values. As an example, when we asked users to write numerical values for two quantitative fields in our metadata form, “TREATMENT_TIME” and “READ_LENGTH”, and specified the expected units (minutes and base pairs, respectively), we still observed entries in hours or days, or in which the units were written, e.g. “50 bp”, which notably complicates the parsability of the metadata. Even fields where free text is accepted, e.g. a description of the sequencing experiment in the words of the experimentalist, we often see typos, inconsistency across experiments and incompleteness.

3. Who collects the metadata? We recommend that the experimentalists fill in the metadata form because, first, they know their experiments the best and, second, because it shares the burden of collecting the metadata and speeds it up. There may be certain resistance from the experimentalists to adopt or change to a metadata collection system because it requires time from them and they may not see the long run benefits. Making the presence of the metadata mandatory for the analysis of the sequencing data is very persuasive. In addition, we recommend that the data manager validates the metadata and update them as needed; we prefer that editing the metadata is restricted to the data manager to minimize the chances of corrupting the information.
4. When are metadata collected? We insist in collecting the metadata always before the analysis of the raw data starts. If metadata are integrated with the analysis of the sequencing data, several fields may be needed to process the data. Even for metadata that are only collected for the sake of describing the experiment, early collection increases the accuracy and completeness of the information.

Altogether, implementing the metadata collection system has a high cost in terms of human time but the maintenance cost is moderate, with experimentalists collectively providing the metadata and the data manager validating them.

Sample identification

The implementation of a system to uniquely identify sequencing samples as presented in the **Additional file 1b** has moderate costs for experimentalists, analysts and the data manager as it requires (i) defining which fields unambiguously identify a sequencing sample and (ii) write a program that converts metadata values into the unique sample identifier. On the other side, implementing a simple auto-incremental sample identification system has virtually zero cost in terms of time and is effective as well. In both cases the cost of maintenance is very low as the data manager only has to name samples and the associated files as they come.

Data and analysis organization

We acknowledge that an effective organization of the data needs to be adapted to the needs of the group or project. In our case, we store raw files in dated directories because we receive sequencing runs over time; we generate a directory for the processed data of each sample because these may be used by independent projects; and we group results by user and analysis thread because we provide support to several users each working on several interconnected projects (**Figure 1b**). While our approach may work elsewhere, we recommend that each group or project deeply plans how data are received, processed and shared in order to define its own organisation. Implementing a data organisation plan is costly in terms of time, mostly for the data manager, but the maintenance cost is minimal.

Documentation

The cost of documenting how data is processed is high and shared by the data manager and analysts.

Reproducibility via virtual machines

Virtual machines allow developers and analysts to ensure that their results are reproducible on different hardware, and they greatly simplify the problem of installing dependencies and external libraries.

Systems like Docker and Docker Hub make it easy to share such virtual environments with the community. However, virtual machines have limitations and costs that should be considered.

1. How long should the machine run? Virtual machine systems like Docker evolve fast, and they tend to not be backward-compatible. Maintaining an analysis running in a virtual environment for more than two years requires to check and update the virtual machine on every new release of the system. Virtual machines have to be viewed as very expensive long term storage.
2. What is the purpose of setting up a virtual machine? The maintenance time required for running virtual machines has to be factored in. It is less expensive to run analyses and store the results on a local computer cluster than on a virtual machine. If the analyses are not meant to be shared with the community, there may be no need for virtual machines at all.
3. Is opacity an issue? Virtual machines are not a trump against code documentation. The good practice for code documentation applies equally in virtual environment, so the cost of writing readable code is in general not reduced by using virtual machines.

Autonomy of the experimentalists

The biggest cost of putting tools for the interactive visualisation of the data into place is their implementation by analysts and the data manager who develop them. Even with availability of tools like R Shiny, notable coding is needed for each feature that is added. Therefore, we think that such tools need to be tailored to the needs of the users.

Bibliography

1. Wilkinson MD, Dumontier M, Aalbersberg IJJ, Appleton G, Axton M, Baak A, et al. The FAIR Guiding Principles for scientific data management and stewardship. Sci Data. 2016;3:160018. doi:10.1038/sdata.2016.18.
