# Supplementary figures and images for: Parallel sequencing lives, or what makes large sequencing projects successful
Source: Gigascience. 2017 Oct 18;6(11):1–6. doi: 10.1093/gigascience/gix100 (PMC5714127; doi:10.1093/gigascience/gix100)

# Add. file 1

a

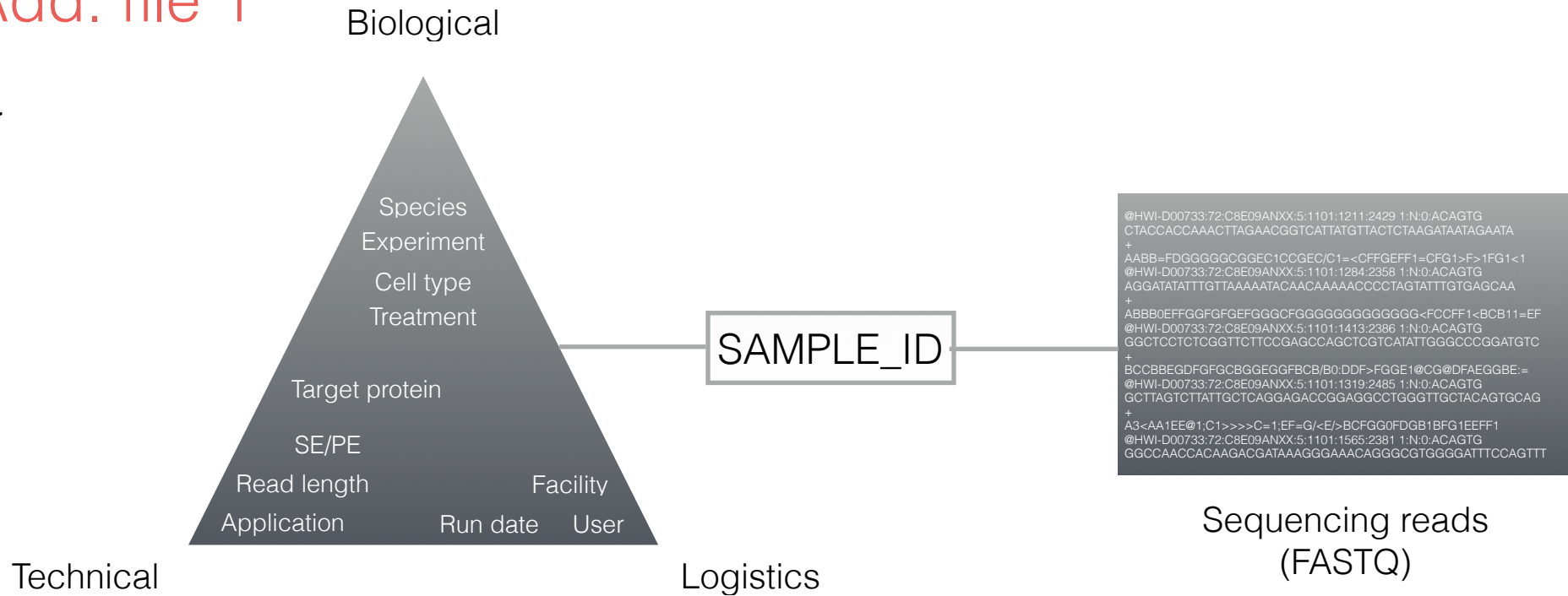

b

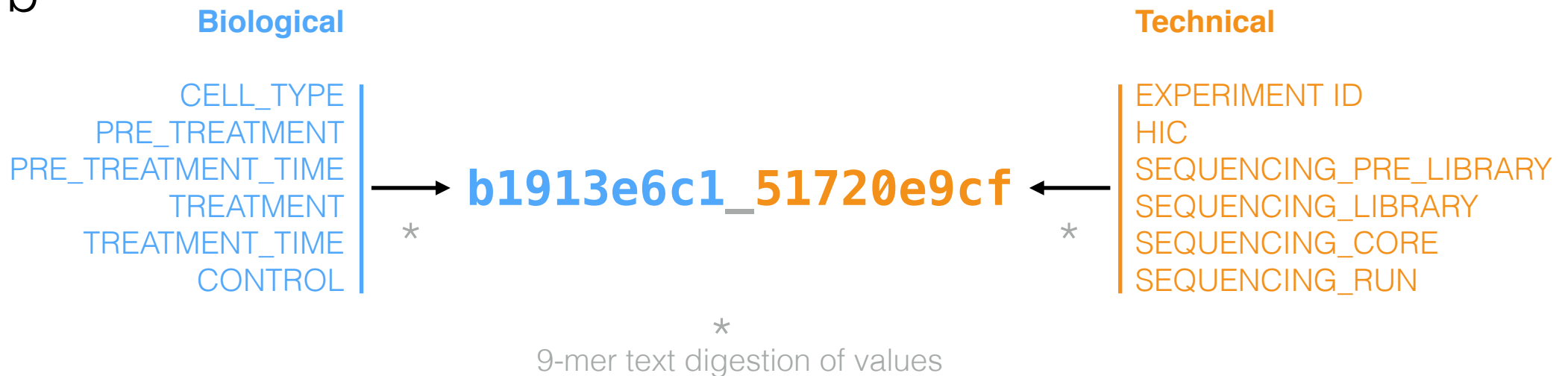

Supplement: Additional Files [file gix100_supp.zip › additional_file01.pdf]

# Add. file 2

a

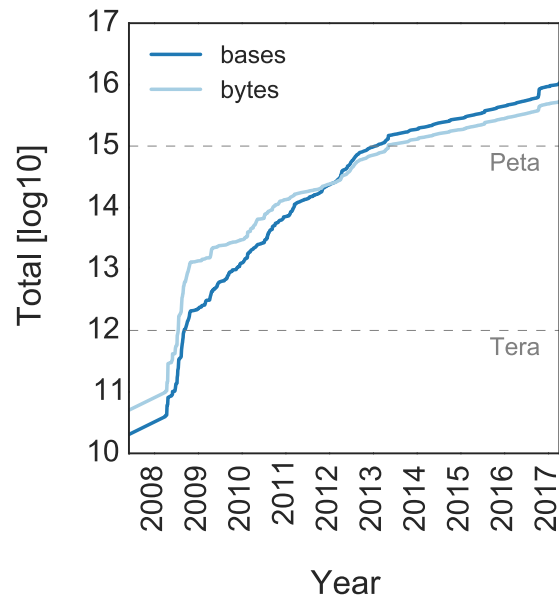

b

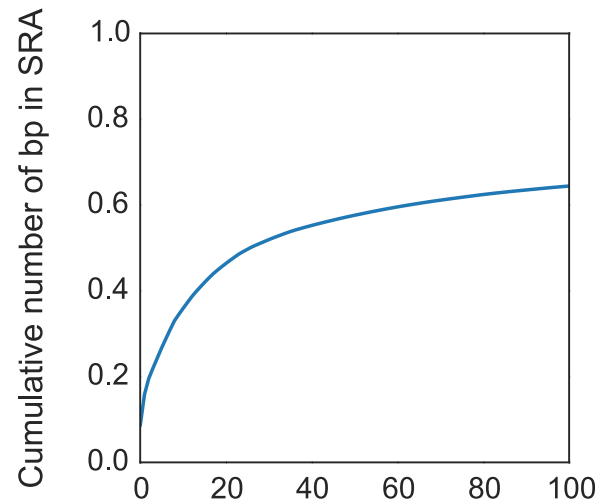

Top 100 contributors  
(out of 90,962 Study Accession IDs in the SRA)

c

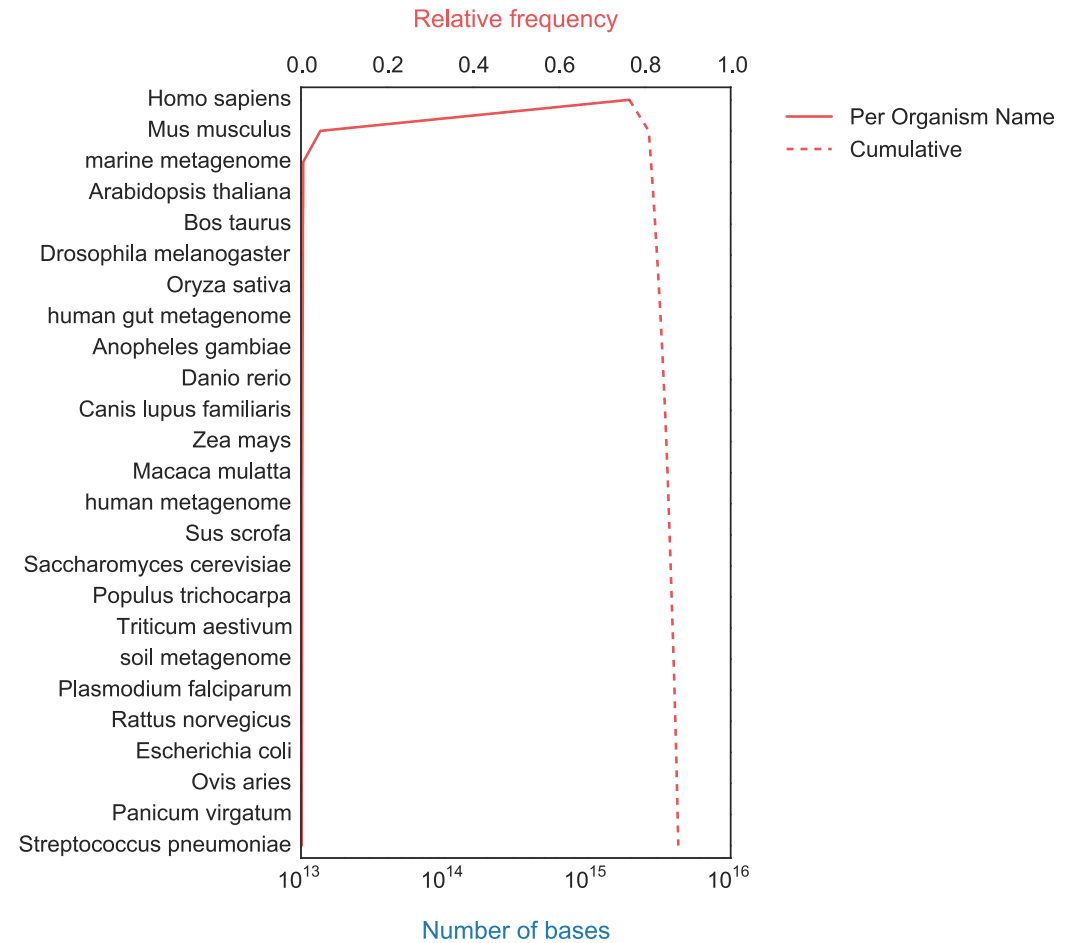

Supplement: Additional Files [file gix100_supp.zip › additional_file02.pdf]

Fig. 1

ONLINE

CLUSTER

a

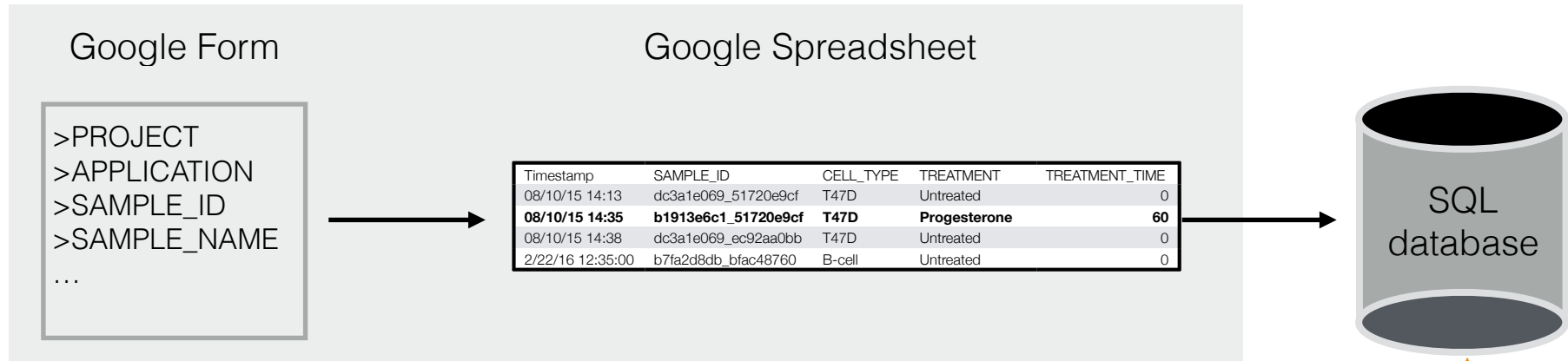

b

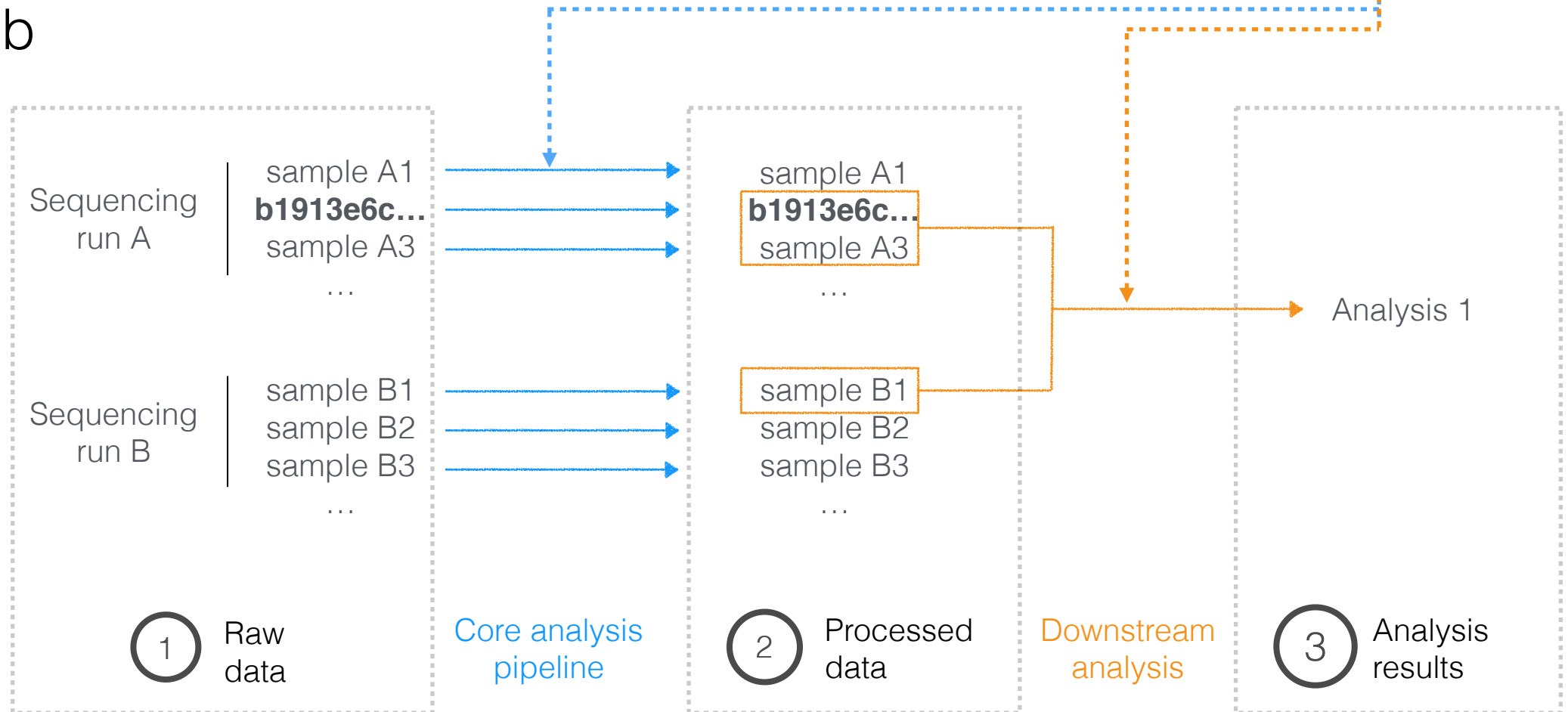

Supplement: Additional Files [file gix100_supp.zip › figure01.pdf]

# Fig. 2

a

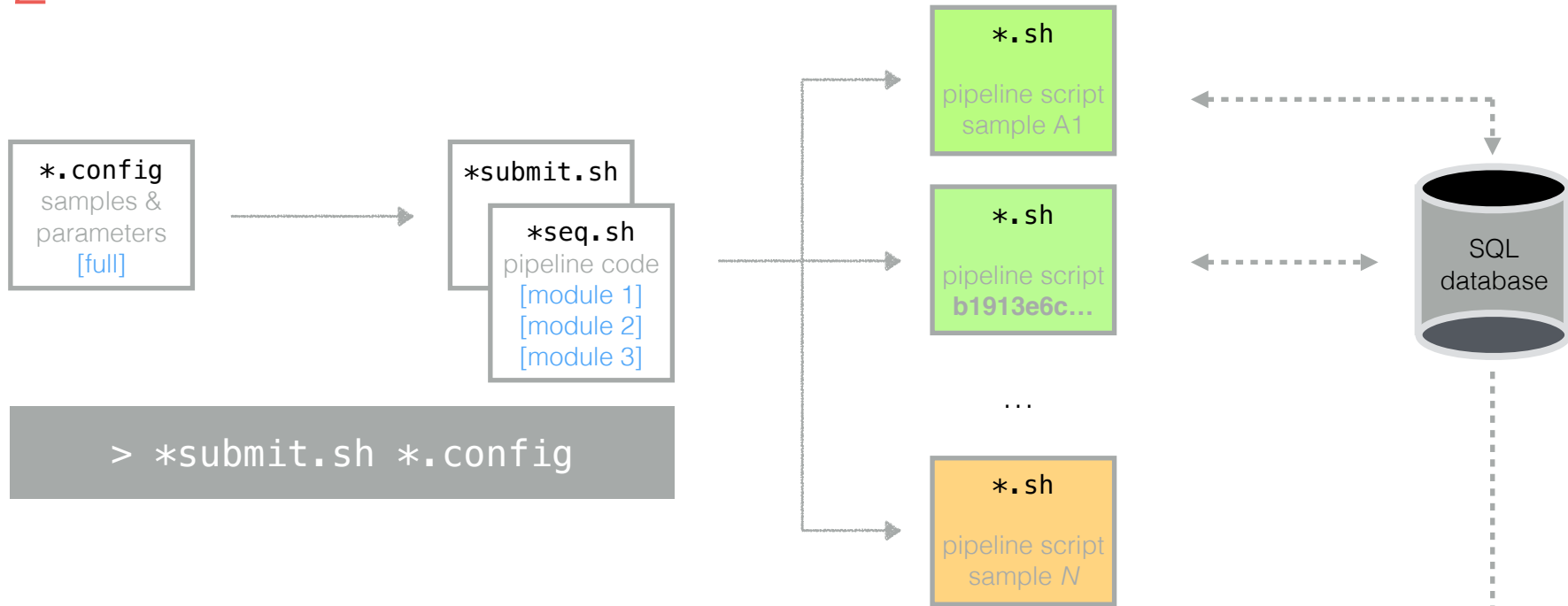

b

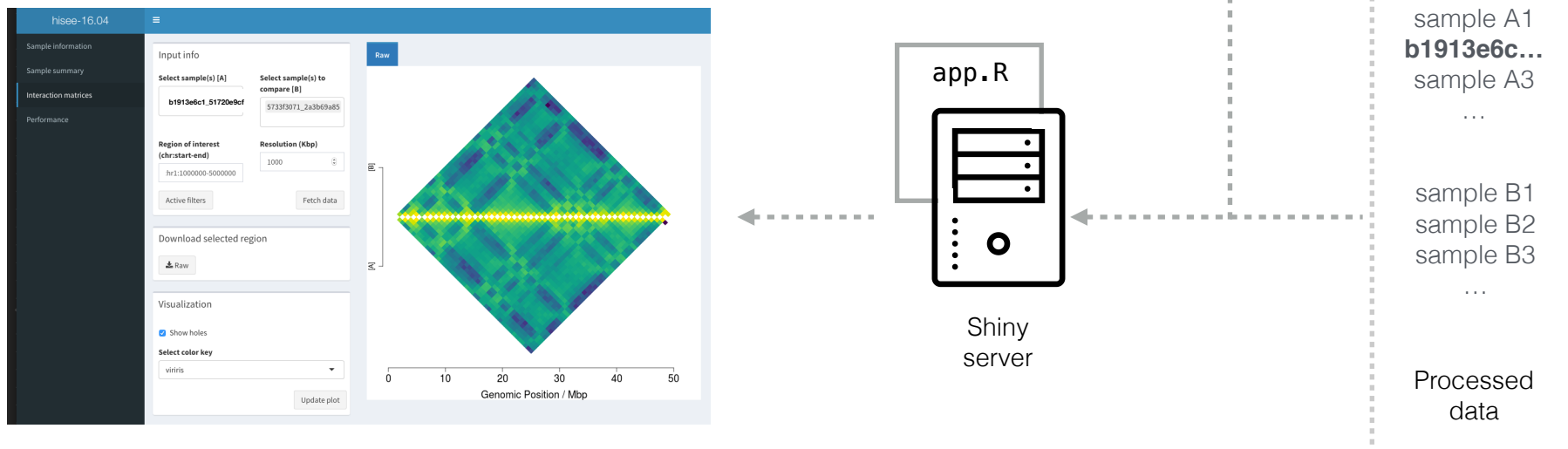

Supplement: Additional Files [file gix100_supp.zip › figure02.pdf]
