# Supplementary material for: Parallel sequencing lives, or what makes large sequencing projects successful
Source: Gigascience. 2017 Oct 18;6(11):1–6. doi: 10.1093/gigascience/gix100 (PMC5714127; doi:10.1093/gigascience/gix100)
Supplement: Additional Files [file acceptance_letter.pdf › acceptance_letter.pdf]

Javier Quilez
CRG-Centre for Genomic Regulation
C/ Dr. Aiguader, 88, PRBB Building
08003 Barcelona, Spain
Tel. +34 93 316 01 15
javier.quilez@crg.eu

September 29, 2017

Dear Editor,

We are very pleased that our manuscript is accepted for publication in GigaScience.

We have incorporated your editorial suggestions into the enclosed version of the *"Parallel sequencing lives, or what makes large sequencing projects successful"* manuscript. In addition, the "Didactic dataset" GitHub repository is now licenced under the MIT Licence (https://github.com/4DGenome/parallel_sequencing_lives/blob/master/LICENSE). We further reviewed the manuscript with the aim to shorten it, but we are afraid we cannot reduce the text without substantially affecting the message, clarity and readability of the story.

We confirm that all the authors have approved the revised manuscript for submission.

Please do not hesitate to contact me if you require any further details.

Sincerely,

Javier Quilez, PhD

Bioinformatician, Gene Regulation, Stem Cells and Cancer Programme
CRG-Centre for Genomic Regulation
